# Male hatchling production in sea turtles from one of the world’s largest marine protected areas, the Chagos Archipelago

**DOI:** 10.1038/srep20339

**Published:** 2016-02-02

**Authors:** Nicole Esteban, Jacques-Olivier Laloë, Jeanne A. Mortimer, Antenor N. Guzman, Graeme C. Hays

**Affiliations:** 1 Swansea University, Department of Biosciences, Swansea, SA2 8PP, United Kingdom; 2 University of Florida, Department of Biology, Gainesville, FL 32611, United States of America; 3 US Naval Facilities Engineering Command Far East, Public Works Department, Diego Garcia, FPO AP 96595, British Indian Ocean Territory; 4 Deakin University, Geelong, Centre for Integrative Ecology (Warrnambool campus), Victoria, Australia

## Abstract

Sand temperatures at nest depths and implications for hatchling sex ratios of hawksbill turtles (*Eretmochelys imbricata*) and green turtles (*Chelonia mydas*) nesting in the Chagos Archipelago, Indian Ocean are reported and compared to similar measurements at rookeries in the Atlantic and Caribbean. During 2012–2014, temperature loggers were buried at depths and in beach zones representative of turtle nesting sites. Data collected for 12,546 days revealed seasonal and spatial patterns of sand temperature. Depth effects were minimal, perhaps modulated by shade from vegetation. Coolest and warmest temperatures were recorded in the sites heavily shaded in vegetation during the austral winter and in sites partially shaded in vegetation during summer respectively. Overall, sand temperatures were relatively cool during the nesting seasons of both species which would likely produce fairly balanced hatchling sex ratios of 53% and 63% male hatchlings, respectively, for hawksbill and green turtles. This result contrasts with the predominantly high female skew reported for offspring at most rookeries around the globe and highlights how local beach characteristics can drive incubation temperatures. Our evidence suggests that sites characterized by heavy shade associated with intact natural vegetation are likely to provide conditions suitable for male hatchling production in a warming world.

Global climate change continues at an unprecedented rate: each of the last three decades has been successively warmer at the Earth’s surface than any preceding decade since 1850, and global temperatures have increased by 0.85 °C from 1880 to 2012^1^. Animals and plants are responding to these thermal changes in a number of ways, including changes in distribution, abundance and phenology^e.g.^^2^,^3^,^4^,^5^. Climate change impacts may be pronounced, and are of high conservation concern, in species exhibiting environmental sex determination (ESD), the most common form of which is temperature-dependent sex determination (TSD) where the temperatures exhibited during embryonic development determine the sex of the offspring. Even slight changes in temperature (1–2 °C) during the temperature-sensitive period (TSP) may alter offspring sex ratios of species with TSD, often determined by mean nest temperatures during the middle third of incubation^5^ or by a complex effects of thermal diel fluctuations^6^. TSD applies to the majority of reptiles^7^ and some fish^8^.

Sea turtles exhibit TSD, typically with a pivotal incubation temperature of 29 °C so that eggs incubating below this temperature produce a majority of males and eggs incubating at warmer temperatures produce a majority of females^9^,^10^. TSD causes primary sex ratios to vary within clutches^11^, among beaches^12^, as well as during the course of a season^13^,^14^. Studying the temperatures of incubating nests has proven to be informative and useful for studying population viabilities in the context of a warming world^15^,^16^ and is key to understanding whether or not turtle populations are threatened by higher temperatures. Of particular concern is the observation that highly female biased hatchling production seems to dominate in sea turtles and the circumstances that may produce male biased hatchling production remain equivocal. Against this background incubation conditions were examined for turtles nesting on Diego Garcia, the largest island of the Chagos Archipelago (Western Indian Ocean). This site lies within one of the World’s largest marine protected areas^17^ which affords nesting turtles total protection. The archipelago was classified as a globally significant turtle breeding site^18^ for both the endangered green turtle, *Chelonia mydas*^19^ and critically endangered hawksbill turtle, *Eretmochelys imbricata*^20^. The protection offered by the marine protected area is likely to further enhance the global significance of these populations over time. Many of the nesting beaches of Chagos are well shaded having retained their dense supra-littoral natural vegetation associations. The dense shade, combined with narrow beach platforms which require turtles to lay clutches near the sea, and heavy seasonal rainfall together predict relatively cool incubation temperatures. This study assesses sand temperatures at sea turtle nest depths and estimates likely hatchling sex ratios for this site. These data are then compared to similar data collected at nesting sites elsewhere in the World and in this way we provide a conceptual framework for identifying which sea turtle nesting beaches are likely to suffer most acutely from warming in future decades.

## Results

**Figure 1:**
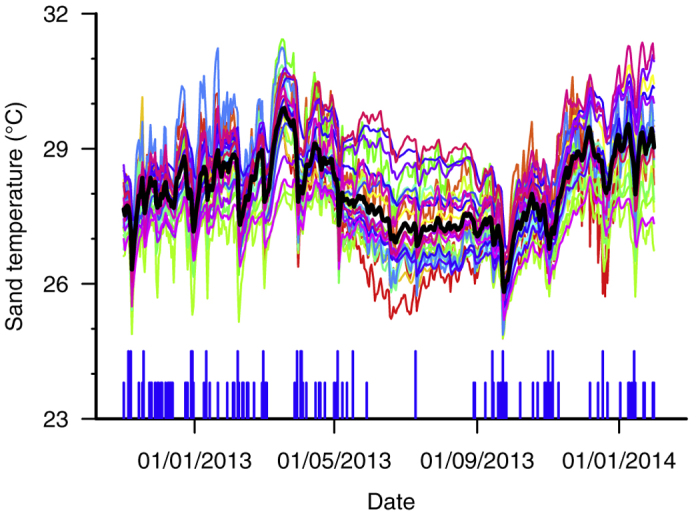
Sand temperatures recorded on Diego Garcia between 16 October 2012 and 6 February 2014 at nest depths (30–80 cm). Each coloured line represents one of the 29 temperature loggers deployed and the black line represents the mean for all loggers. The short and long vertical blue lines indicate days for which precipitation was >10 mm and >50 mm respectively. Variation between loggers can be explained by the different beach zones and different depths at which the loggers were buried.

Twenty-nine loggers were recovered with each logger providing data for up to 457 days. One logger was lost due to erosion and one logger stopped logging prematurely due to battery failure. In total 12,546 days of sand temperature data were obtained. The loggers placed at depths of 30 cm (N = 8) and 50 cm (N = 9) were used to estimate conditions in hawksbill nests; while those at 50 cm (N = 9), 70 cm (N = 9), and 80 cm (N = 3) were used to represent green turtle nests. Sand temperature at all sites showed similar trends in seasonal variation: increasing in October-March during the austral spring and summer, broadly straddling the pivotal temperature, and decreasing from April-September during the austral winter (Fig. 1). Sudden and large decreases in temperature (>1.5 °C within 24 hours) were consistent across all sites and depths and coincided with rainfall events, particularly during the wetter northwest monsoon season (December–March) and, to a lesser extent, during the drier southeast monsoon (April–October) (see Supplementary Fig. S1).

**Figure 2:**
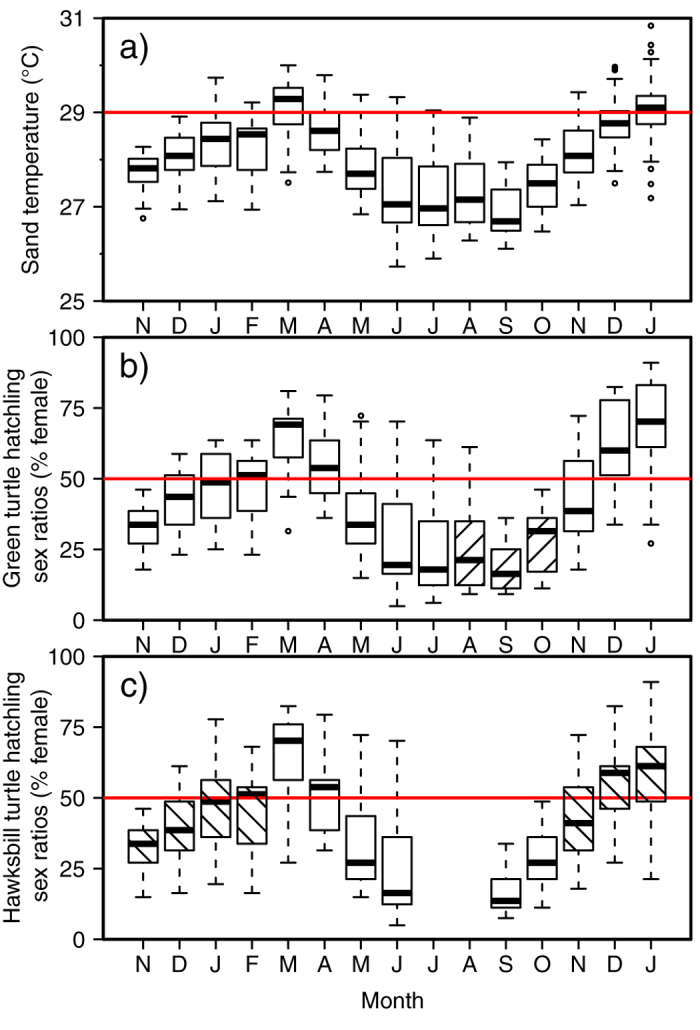
The mean monthly sand temperature for each of 29 loggers on Diego Garcia from November 2012 until January 2014 (excluding the months for which any days of data were missing) and the reconstructed primary sex ratios. (**a**) Seasonality is shown here with lower temperatures during the months of June-October and higher temperatures during the months of December–April. The horizontal red line defines the pivotal temperature of 29 °C. (**b**) Green turtle hatchling production is male biased through the majority of the year. Green turtles nest throughout the year with the peak incubation period (stippled) coinciding with the coolest months of the year. (**c**) Hawksbills nests incubate during the months of September to June with peak incubation period (stippled) straddling the 1:1 male to female hatchling sex ratio. The horizontal line indicates the median temperature; boxes define upper and lower quartiles and whiskers show the data range; individual circles represent outliers of mean monthly temperature recorded for individual loggers.

Mean monthly temperatures were calculated for each logger, excluding those months for which any days of data were missing, and then used to describe annual seasonality. Seasonality is clearly evident, with mean monthly sand temperatures exceeding 28 °C in December–April during the austral summer (mean temperature range of 28.1–29.1 °C) and staying below 27.5 °C in June–October during the austral winter (mean temperature range of 26.9–27.5 °C) (Fig. 2a).

**Figure 3:**
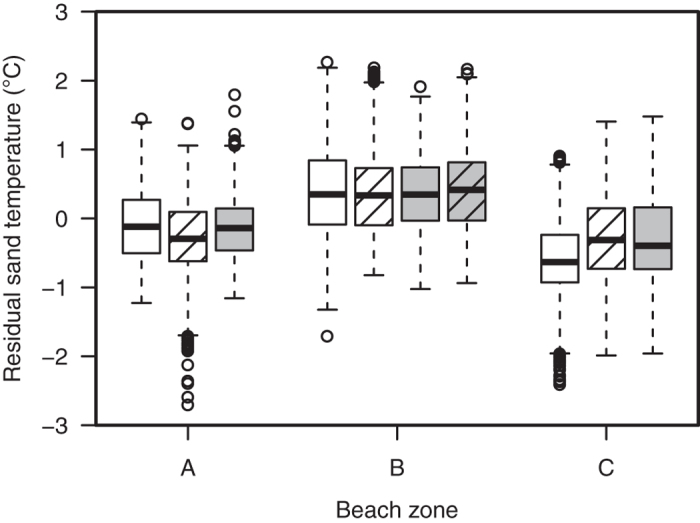
Residual sand temperatures for each beach zone. Loggers of all sites are pooled together by beach zone and by depth (30 cm: white boxes; 50 cm: stippled white boxes, 70 cm: grey boxes; 80 cm: stippled grey box). The loggers placed in the sites partially shaded in vegetation above the Spring HWL (**B**) recorded warmer temperatures than the loggers in the sites partially shaded on the open beach at the Spring HWL (**A**) or in the sites heavily shaded in the vegetation above the Spring HWL (**C**) over the entire study period (χ^2^ = 3688.8, df = 2, *p* < 0.001). The horizontal lines indicate the median temperature; boxes define upper and lower quartiles and whiskers show the data range; individual circles represent outliers.

For further analyses, the effect of seasonality was removed by subtracting the mean sand temperature for all loggers (black line in Fig. 1) from each individual logger, and using a simple moving average (N = 10 consecutive daily means) to smooth the data. The resulting dependent variable is labelled “residual sand temperature”. A linear mixed effects analysis of the relationship between residual sand temperatures, depth and beach zone was performed. Depth and beach zone were entered as fixed effects (without interaction terms) and site entered as a random effect. Visual inspection of residual plots did not reveal any obvious deviations from homoscedasticity or normality. P-values were obtained by likelihood ratio tests. Residual sand temperatures were different at each beach zone and at different depths (Fig. 3). Beach zone affected residual sand temperature (χ^2^ = 3689, df = 2, *p* <0.001) with the warmest temperatures found in the sites partially shaded in vegetation above the Spring High Water Line (HWL). Mean residual sand temperatures were 0.64 °C (s.e.m. = 0.01) cooler at the sites partially shaded on the open beach at the Spring HWL and 0.75 °C (s.e.m. = 0.01) cooler in the sites heavily shaded in the vegetation above the Spring HWL. Relative to the effect of beach zone or seasonality on sand temperatures, the effect of depth was only minor. Depth affected residual sand temperature (χ^2^ = 142.2, df = 3, *p* <0.001) with the shallow depth being the coolest with the mean difference between the shallowest and the deepest loggers being 0.14 °C (s.e.m. = 0.01). Further analyses reveal that the effect of depth is not constant through the seasons and that shallow temperatures are cooler relative to deeper temperatures only during cooling periods. This relationship is inversed during periods of warming (see Supplementary Fig. S1). The maximum difference between the shallowest and the deepest logger during a period of warming is 0.46 °C.

For each species, hatchling sex ratios were reconstructed for the study period assuming a homogenous distribution of nests across sites, beach zones, and depths (using data from 30, 50 and 70 cm depths). The data show that hatchling sex ratios vary through the year (Fig. 2b,c). For example in March reconstructed sex ratios for hawksbills and green turtles, respectively, were 64% and 65% female; but they changed to 17% and 19% female, respectively in September. Peak nesting for green turtles occurs during some of the coolest months of the year (in the austral winter) whereas peak nesting for hawksbills occurs from October to January during warmer months (in the austral summer). It follows that hatchling sex ratios for green turtles tend to be male-biased and those of hawksbills more balanced. Assuming that hawksbill^21^ and green^22^ turtle nesting seasonality is similar to that documented in Seychelles, the nearest site for which data exist, then in the course of an entire year overall male hatchling production for hawksbills and green turtles, respectively, would have been 53% and 63%.

**Figure 4:**
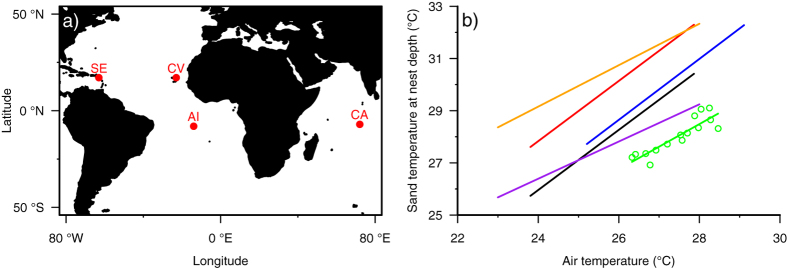
Mean sand temperature versus mean air temperature for rookeries in the Atlantic, Caribbean and Indian Ocean where the same methodologies have been employed. (**a**) Locations of sites in: the Caribbean–St Eustatius (SE); the Atlantic--Ascension Island (AI) and Cape Verde (CV); and the Indian Ocean–Chagos Archipelago (CA). This figure has been created in R^57^ using the Rworldmap package^59^. (**b**) Each point for Chagos Archipelago (in green) represents a mean monthly sand temperature value recorded across all loggers combined. The least squares fit regression equation for Diego Garcia is: mean monthly sand temperature = 0.87 x mean monthly air temperature +4.25. Regression lines are shown for: the dark-coloured beaches (in orange) and light-coloured beaches (in purple) of Ascension^12^; the dark-coloured beaches (in red) and light-coloured beaches (in black) of Cape Verde^29^; and St Eustatius^14^ (in blue).

Mean monthly sand temperature correlated with mean monthly air temperature (R^2^ = 0.79, F_1,13_ = 48.79, *p* < 0.001). A comparison of data from similar studies in the Caribbean and Atlantic, demonstrates that while sand temperatures at nest depth increase with air temperature across all sites, the intercepts of these relationships were quite different (Fig. 4b). This finding was confirmed by an ANCOVA (F_4,585_ = 448.6, *p* < 0.001). So for example, at an air temperature of 27.0 °C, the mean temperature at nest depths was 30.8 °C at St Eustatius (Caribbean), 29.4 °C and 31.3 °C on the light and dark beaches on Cape Verde, and 28.5 °C and 31.5 °C on the light and dark beaches at Ascension Island. The relationships between sand and air temperatures were broadly similar across sites, with a 1 °C increase in air temperature producing an increase of between 0.71–1.15 °C in mean sand temperature at nest depths.

## Discussion

The correlation between warm incubation temperatures and female-biased hatchling production has been widely reported in sea turtle populations^23^, heightening concerns that climate warming may be deleterious to sea turtles and ultimately lead to the production of female only populations and eventual extinction. For example, in a comparison of 28 loggerhead turtle nesting sites around the world it was recently reported that 19 sites produced >70% female hatchlings^23^. A similar tendency for production of female hatchlings has also been reported for other species, for example for green turtles nesting on Ascension Island (central Atlantic)^24^, for hawksbills and green turtles nesting at St Eustatius (Caribbean)^14^ and for loggerheads nesting in Greece^25^ (Mediterranean).

In contrast, relatively few sites have reported mean sand temperatures below 29.0 °C at nest depths indicative of male hatchling production. Exceptions include loggerhead turtles nesting in southern Brazil^26^ and North Carolina USA^27^, and leatherbacks nesting in Papua New Guinea^28^. It follows that the relatively low sand temperatures at nest depths recorded at Diego Garcia, and the likelihood that they would produce balanced sex ratios amongst turtle hatchlings, are noteworthy. We predict a range of demographic consequences for these balanced hatchling sex ratios. For example we might expect more male biased operational (breeding) sex ratios, given that male turtles are thought to breed more frequently than females^23^. It is interesting that green turtle nesting occurs year-round in Chagos similar to other rookeries in the Western Indian Ocean^21^. Overall there is thought to be an appreciable number of nesting females each year (circa 600^18^), but the nesting density is fairly low as it is scattered across some 55 islands in the archipelago. This combination of diffuse low density nesting and a protracted nesting season might, with highly female biased operational sex ratios, be expected to lead to low male-female encounter rates and implications for clutch fertility and genetic diversity. However our inference of high ratios of breeding males produced at Diego Garcia in turn likely means that clutch fertility is unlikely to be compromised despite a relatively low density of nesting females.

While it is clear from the empirical observations that relatively cool sand temperatures occur at Diego Garcia, understanding the drivers of these conditions is important as it may help identify ways in which climate warming impacts may be mitigated in order to produce both male and female hatchlings in the future^29^. It has been muted that turtles might lay deeper nests to combat future warming. Nest depth is probably a consequence of female size and in particular her ability to reach down with her rear flipper when excavating a nest chamber^30^. Hence it is not simple for turtles to adjust their nest depths. Our results show, however, that depth had relatively little impact on sand temperature across the range of depths examined, with the mean difference between deep loggers and shallow loggers being only a fraction of a degree. Similarly elsewhere fairly limited impact of depth on sand temperatures have been noted^14^,^31^,^32^. We also report that the effect of depth changes during warming and cooling periods, with surface temperatures always being more extreme than deep temperatures: sand temperature changes most appreciably with depth near the surface and depth variations become dampened down with increasing depth. So, for example, for shallow nesting species such as freshwater turtles and small sea turtles (e.g. mean nest depth of 45.0 cm reported for both olive ridleys^33^ and hawksbills^34^), depth effects on incubation temperature may be more profound. This may explain why hawksbill turtles typically prefer to nest under vegetation even at sites where open sand is readily available to them^35^,^36^. Shade from the vegetation may stabilize sand temperature in shallow nests. Differences in microhabitat selection on nesting beaches among sea turtle species are often reported^37^,^38^ and should be considered across the Chagos archipelago in future.

For both the deep nesting green turtles, and the shallower hawksbills, sand temperature was impacted by both location of the nest on the beach platform and day of the year. These effects are likely to be the intrinsic drivers of the heat balance of the sand. Warmer air temperatures and increased solar radiation will lead to more heat propagating downwards through the sand and hence warmer temperatures at nest depths. Indeed heat transfer models of soils can be used to predict temperature at depth directly from such factors^39^. Similarly, shaded nests and those closer to the sea are expected to be cooler^36^,^38^,^40^,^41^, both predictions confirmed by our results. Such seasonal and nest positioning effects have been noted before. For example, Mrosovsky^13^ was one of the first to show marked seasonal changes in hatchling production on the same turtle beach with warmer female-producing temperatures at the height of the summer and cooler male-producing temperatures in the spring and autumn. This finding has since been noted several times^14^,^42^. Yet the sand conditions at Diego Garcia are relatively cool despite the equatorial location of the island and resulting warm air temperatures. For example, at the Cape Verde Islands and at St. Eustatius equivalent air temperatures during the breeding season produce warmer temperatures at nest depths. The most parsimonious explanation for this finding is that at Diego Garcia the combination of shading, rainfall induced cooling and clutches being laid relatively close to the sea due to the narrow beaches produces this overall tendency for cooler incubation conditions.

This conclusion supports those reached from other studies across the globe^38^,^41^,^43^,^44^ and highlights how profoundly important shade may be for cooling nest temperatures at a site. This general conclusion indicates that maintaining native dune vegetation and planting appropriate species of trees and shrubs behind nesting beaches may be among the best strategies to mitigate the effect of warming temperatures at a turtle rookery. Such conservation practices have already proven successful^44^ and provide the additional benefit that the presence of trees along beaches can also block disruptive artificial light glows that disorient both nesting females and hatchlings^45^. However, despite the benefits offered by vegetation, a range of negative effects has also been reported^46^,^47^,^48^ so any manipulation of vegetation on nesting beaches needs to be done with caution. Alternative strategies for cooling sand include sprinkling sand with water^33^,^49^ or relocating nests to a controlled hatchery^50^.

Protracted rainfall was previously known to be a driver of sand temperature^51^ but its importance may have been overlooked at other sites. This is of particular interest since climate change will not only bring about changes in air temperatures, but also changes in other environmental variables such as precipitation and storm frequency. The comparison of different sea turtle nesting sites conducted in the present study shows that while air temperature is an important driver of sand temperature at nest depths, there is no single relationship between sand and air temperature that can accurately predict sand temperature from air temperature alone. Hence a global assessment of the implications of climate warming for sea turtle hatchling sex ratios will need further information beyond predicted changes in air temperature. At sites lacking direct measurement of sand temperature, soil models that predict the sand temperature from a suite of abiotic drivers (e.g. air temperature but additionally other factors including wind speed, sand albedo, shading and soil conductivity) may be possible^39^. Future studies should endeavour to assess hatchling sex ratios across broader areas that encompass the entire nesting range of populations, as well as assessing how hatchling sex ratios translate through the lives of turtles into operational sex ratios realised on the mating grounds.

Sea turtles have the potential to adapt to warming sand temperatures by adjusting their nesting seasons^52^. At Diego Garcia, seasonality of sand temperatures suggests that if turtles were to shift their nesting seasons to cooler months of the year this could theoretically mitigate some of the negative aspects of warmer sand temperatures. If this is possible in practice it will depend largely on the magnitude and rapidity of warming temperatures and whether or not turtles will have the time to adapt to these changes.

In conclusion, empirical measurements of sand temperatures at nest depths at Diego Garcia, Indian Ocean, highlight a breeding site for hawksbill and green turtles that currently produces a fairly balanced sex ratio of hatchlings, most likely due to shading, rainfall-induced cooling and nests being laid relatively close to the sea. As such, incubation temperatures at this site are likely to be fairly resistant to warming predicted to occur with climate change.

## Methods

Temperature loggers (Tinytag Plus 2 model TGP-4017, Gemini Data Loggers, UK, dimensions 34 × 51 × 80 mm and weighing 110 g, accurate to <0.5 °C) recorded sand temperature every 4 h at nest depths on a beach used by nesting hawksbill and green turtles on Diego Garcia (07°18′ S, 72° 24′ E) within the Chagos Archipelago (Indian Ocean). The atoll of Diego Garcia hosts the highest nesting density of hawksbills and green turtles in Chagos^18^. The study beach is 2.75 km and is located on the south-eastern coast of the main island. It was identified as an index nesting beach for green and hawksbill turtles during previous surveys^18^, though scattered nesting occurs in similar habitat along the 60 km oceanic coastline of the main island of Diego Garcia.

The impact to natural conditions during burial of loggers was minimized by excavating a sand core and then replacing it back on top of the logger. This was achieved by hammering a 125 mm diameter plastic pipe to the desired depth of the logger, covering it to create a vacuum and then removing the pipe full of sand. The depth of the hole was verified using a semi-rigid tape measure, after which the logger was dropped into the hole. The sand was then emptied out of the pipe onto the logger. A string was connected to the logger to facilitate its relocation.

In contrast to some other sites in the World where beaches are wider or lack supra-littoral vegetation, the beaches of Diego Garcia offer little open sand for nesting. The Spring HWL is typically very close (<1 m) to the vegetation. There is a littoral hedge of native Indo-Pacific shrub *Scaevola taccada,* scattered coconut palms *Cocos nucifera,* as well as other trees and shrubs^53^, particularly *Suriana maritima* and heliotrope *Argusia argentea*. Turtles usually nest within the vegetation, with almost all nest sites subjected to heavy or partial shading. To represent the range of nest sites available to turtles, loggers were buried in three zones: sites heavily shaded in the vegetation above the Spring HWL, sites partially shaded in vegetation above the Spring HWL, and sites partially shaded on the open beach near the Spring HWL. Three sites were selected along the index nesting beach based on historical nesting activities (indicated by presence of body pits): site 1 was 110 m from site 2 and site 2 was 50 m from site 3. A total of 30 loggers were buried. In each beach zone at each site loggers were buried at 30 cm, 50 cm, 70 cm. A single further logger was buried at 80 cm to represent the extreme depth for green turtles in all sites partially shaded in vegetation above the Spring HWL. These depths straddle the range of nesting depths recorded for hawksbills (30–45 cm)^34^ and green turtles (70–85 cm)^43^. Loggers were buried 1 m apart in each beach zone.

Air temperature data for the Indian Ocean basin was obtained from the International Comprehensible Ocean-Atmosphere Data Set (ICOADS) through the National Center for Atmospheric Research (NCAR)^54^. Due to the relative isolation of Chagos and consequent low number of historical data points in the vicinity of Diego Garcia, a 10° by 10° area extending from 2–12 °S and 66–76 °E was used. The Enhanced ICOADS Monthly Summary Release 2.5 at 2-degree spatial resolution was used. Visual inspection showed that air temperatures within the selected area were broadly homogenous and so the exact area used in this analysis did not impact our overall conclusions. Historical rainfall data collected at Diego Garcia Airport (obtained from Weather Underground^55^) is representative of that at the nesting beach study site located 13 km away.

Primary sex ratios were estimated using the relationship between incubation temperature and hatchling sex ratios^29^. A value of 0.5 °C was assumed to be representative of metabolic heating, in line with values reported at nesting sites across the world^56^, and a pivotal temperature of 29 °C was used as illustrative for all species of sea turtles^57^. Loggers buried at 50–70 cm were used to reconstruct the hatchling sex ratios of green turtles and loggers buried at 30–50 cm for the hawksbills. Mean annual hatchling sex ratio was calculated^23^ for each species using the nesting seasonality of each species from the nearest site for which data exist for hawksbill^21^ and green^22^ turtles and our mean monthly sand temperatures recorded at the respective depths for each species.

Published data by the authors of this paper, using the same methodologies in the Atlantic^12^,^29^ and Caribbean^14^ were used to explore the generalities of drivers behind incubation temperatures.

## Additional Information

**How to cite this article**: Esteban, N. *et al*. Male hatchling production in sea turtles from one of the world's largest marine protected areas, the Chagos Archipelago. *Sci. Rep.* **6**, 20339; doi: 10.1038/srep20339 (2016).

## Supplementary Material

Supplementary Information

## References

[b1] IPCC. Observed changes and their causes In Climate Change 2014: Synthesis Report (eds Pachauri R. K. .) 39–54. (IPCC, 2014).

[b2] Morellet N. . Seasonality, weather and climate affect home range size in roe deer across a wide latitudinal gradient within Europe. J. Animal Ecol. 82, 1326–1339 (2013). 10.1111/1365-2656.12105 23855883

[b3] Lynam C. P. . Have jellyfish in the Irish Sea benefited from climate change and overfishing? Glob. Change Biol. 17, 767–782 (2011).

[b4] Ramp C., Delarue J., Palsbøll P. J., Sears R. & Hammond P. S. Adapting to a warmer ocean—seasonal shift of baleen whale movements over three decades. PLoS One 10, e0121374 (2015). 25785462 10.1371/journal.pone.0121374 PMC4364899

[b5] Lance V. A. Is regulation of aromatase expression in reptiles the key to understanding temperature-dependent sex determination? J. Exp. Zool. 311, 314–322 (2009). 10.1002/jez.465 18668631

[b6] Warner D. A. & Shine R. Interactions among thermal parameters determine offspring sex under temperature-dependent sex determination. Proc. R. Soc. B. 278, 256–265 (2011). 10.1098/rspb.2010.1040 PMC3013385 20685704

[b7] Janzen F. J. & Paukstis G. L. Environmental sex determination in reptiles: ecology, evolution, and experimental design. Q. Rev. Biol. 66, 149–179 (1991). 1891591 10.1086/417143

[b8] Conover D. O. Temperature-dependent sex determination in fishes in Temperature-dependent sex determination in vertebrates (eds Valenzuela N. & Lance V.) 11–20. (Smithsonian Books, 2004).

[b9] Mrosovsky N. Sex ratios of sea turtles. J. Exp. Zool. 270, 16–27 (1994).

[b10] Mrosovsky N. & Pieau C. Transitional range of temperature, pivotal temperatures and thermosensitive stages for sex determination in reptiles. Amphibia-Reptilia 12, 169–179 (1991).

[b11] Hawkes L. A., Broderick A. C., Godfrey M. H. & Godley B. J. Climate change and marine turtles. Endang. Species Res. 7, 137−154 (2009).

[b12] Hays G. C., Broderick A. C., Glen F. & Godley B. J. Climate change and sea turtles: a 150-year reconstruction of incubation temperatures at a major marine turtle rookery. Glob. Change Biol. 9, 642–646 (2003).

[b13] Mrosovsky N. Sex ratio of sea turtles: seasonal trends. Science 225, 739–741 (1984). 17810293 10.1126/science.225.4663.739

[b14] Laloë J.-O., Esteban N., Berkel J. & Hays G. C. Sand temperatures for nesting sea turtles in the Caribbean: implications for hatchling sex ratios in the face of climate change. J. Exp. Mar. Biol. Ecol. 474, 92–99 (2015).

[b15] Saba V. S., Stock C. A., Spotila J. R., Paladino F. V. & Santidrián Tomillo P. Projected response of an endangered marine turtle population to climate change. Nature Clim. Change 2, 814–820 (2012).

[b16] Santidrián Tomillo P., Genovart M., Paladino F. V., Spotila J. R. & Oro D. Climate change overruns resilience conferred by temperature-dependent sex determination in sea turtles and threatens their survival. Glob. Change Biol. 21, 2980–2988 (2015). 10.1111/gcb.12918 25929883

[b17] Cressey D. Uncertain sanctuary. Nature 480, 166–167 (2011). 22158221 10.1038/480166a

[b18] Mortimer J. A. & Day M. Sea turtle populations and habitats in the Chagos Archipelago, British Indian Ocean Territory in Ecology of the Chagos Archipelago (eds Sheppard C. R. C. & Seaward M. R. D.) 159–176 (Linnean Society, 1999).

[b19] Seminoff J. A. *The IUCN Red List of Threatened Species: Chelonia mydas*. (2004) Available at: www.iucnredlist.org/details/4615/0. (Accessed: 5th October 2014)

[b20] Mortimer J. A. & Donnelly M. *The IUCN Red List of Threatened Species: Eretmochelys imbricata*. (2008). Available at: www.iucnredlist.org/details/8005/0. (Accessed: 5th October 2014)

[b21] Mortimer J. A., Camille J.-C. & Boniface N. Seasonality and status of nesting hawksbill (*Eretmochelys imbricata*) and green turtles (*Chelonia mydas*) at D’Arros Island, Amirantes Group, Seychelles. Chelonian. Conserv. Biol. 10, 26–33 (2011).

[b22] Mortimer J. A. Seasonality of green turtle (*Chelonia mydas*) reproduction at Aldabra Atoll, Seychelles (1980–2011) in the regional context of the Western Indian Ocean. Chelonian. Conserv. Biol. 11, 170–181 (2012).

[b23] Hays G. C., Mazaris A. D. & Schofield G. Different male vs. female breeding periodicities help mitigate offspring sex ratios skews in sea turtles. Front. Mar. Sci. 1, 43 (2014).

[b24] Godley B. J., Broderick A. C., Glen F. & Hays G. C. Temperature-dependent sex determination of Ascension Island green turtles. Mar. Ecol. Prog. Ser. 226, 115–124 (2002).

[b25] Godley B. J. . Thermal conditions in nests of loggerhead turtles: further evidence suggesting female skewed sex ratios of hatchling production in the Mediterranean. J. Exp. Mar. Biol. Ecol. 263, 45–63 (2001).

[b26] Baptistotte C., Scalfoni J. T. & Mrosovsky N. Male-producing thermal ecology of a southern loggerhead turtle nesting beach in Brazil: implications for conservation. Animal Cons. 2, 9–13 (1999).

[b27] Hawkes L. A., Broderick A. C., Godfrey M. H. & Godley B. J. Investigating the potential impacts of climate change on a marine turtle population. Glob. Change Biol. 13, 923–932 (2007).

[b28] Steckenreuter A., Pilcher N., Krüger B. & Ben J. Male-biased primary sex ratio of Leatherback turtles (*Dermochelys coriacea*) at the Huon Coast, Papua New Guinea. Chel. Cons. Biol. 9, 123–128 (2010).

[b29] Laloë J.-O., Cozens J., Renom B., Taxonera A. & Hays G. C. Effects of rising temperature on the viability of an important sea turtle rookery. Nature Clim. Change 4, 513–518 (2014).

[b30] Horne C. R. . The effect of thermal variance on the phenotype of marine turtle offspring. Physiol. Biochem. Zool. 87, 796–804 (2014). 25461644 10.1086/678238

[b31] Van de Merwe J., Ibrahim K. & Whittier J. Effects of hatchery shading and nest depth on the development and quality of *Chelonia mydas* hatchlings: implications for hatchery management in Peninsular Malaysia. Austr. J. Zool. 53, 205–211 (2005).

[b32] Van de Merwe J., Ibrahim K. & Whittier J. Effects of nest depth, shading, and metabolic heating on nest temperatures in sea turtle hatcheries. Chel. Cons. Biol. 5, 210–215 (2006).

[b33] Hill J. E., Paladino F. V., Spotila J. R. & Tomillo P. S. Shading and watering as a tool to mitigate the impacts of climate change in sea turtle nests. PLoS One 10, e0129528 (2015). 26030883 10.1371/journal.pone.0129528 PMC4452221

[b34] Hitchins P. M., Bourquin O., Hitchins S. & Piper S. E. Biometric data on hawksbill turtles (*Eretmochelys imbricata*) nesting at Cousine Island, Seychelles. J. Zool. Lond. 264, 371–381 (2004).

[b35] Mortimer J. A. Factors influencing beach selection by nesting sea turtles in Biology and Conservation of Sea Turtles (ed Bjorndal K. A.) 45–51 (Smithsonian Institute Press, 1982).

[b36] Kamel S. J. Vegetation cover predicts temperature in nests of the hawksbill sea turtle: implications for beach management and offspring sex ratios. Endang. Species Res. 20, 41–48 (2013).

[b37] Stoneburner D. L. & Richardson J. I. Observations on the role of temperature in loggerhead turtle nest site selection. Copeia 1981, 238–241 (1981).

[b38] Kamel S. J. & Mrosovsky N. Deforestation: risk of sex ratio distortion in hawksbill sea turtles. Ecol. Appl. 16, 923–931 (2006). 16826992 10.1890/1051-0761(2006)016[0923:drosrd]2.0.co;2

[b39] Fuentes M. M. P. B. & Porter W. P. Using a microclimate model to evaluate impacts of climate change on sea turtles. Ecol. Model. 251, 150–157 (2013).

[b40] Janzen F. J. Vegetational cover predicts the sex ratio of hatchling turtles in natural nests. Ecology 75, 1593–1599 (1994).

[b41] Patino-Martinez J., Marco A., Quiñones L. & Hawkes L. A. A potential tool to mitigate the impacts of climate change to the Caribbean leatherback sea turtle. Glob. Change Biol. 18, 401–411 (2012).

[b42] Godley B. J., Broderick A. C., Frauenstein R., Glen F. & Hays G. C. Reproductive seasonality and sexual dimorphism in green turtles. Mar. Ecol. Prog. Ser. 226, 125–133 (2002).

[b43] Fuentes M. M. P. B., Hamann M. & Limpus C. J. Past, current and future thermal profiles of green turtle nesting grounds: Implications from climate change. J. Exp. Mar. Biol. Ecol. 383, 56–64 (2010).

[b44] Wood A., Booth D. T. & Limpus C. J. Sun exposure, nest temperature and loggerhead turtle hatchlings: implications for beach shading management strategies at sea turtle rookeries. J. Exp. Mar. Biol. Ecol. 451, 105–114 (2014).

[b45] Salmon M. Artificial night lighting and sea turtles. Biologist 50, 163–168 (2003).

[b46] Godfrey M. H. & Barreto R. Beach vegetation and sea finding orientation of turtle hatchlings. Biol. Cons. 74, 29–32 (1995).

[b47] Conrad J. R., Wyneken J., Garner J. A. & Garner S. Experimental study of dune vegetation impact and control on leatherback sea turtle *Dermochelys coriacea* nests. Endang. Species Res. 15, 13–27 (2011).

[b48] Ditmer M. A. & Stapleton S. P. Factors affecting hatch success of hawksbill sea turtles on Long Island, Antigua, West Indies. PLoS One 7, e38472 (2012). 22802928 10.1371/journal.pone.0038472 PMC3389013

[b49] Jourdan J. & Fuentes M. M. P. B. Effectiveness of strategies at reducing sand temperature to mitigate potential impacts from changes in environmental temperature on sea turtle reproductive output. Mitig. Adapt. Strateg. Glob. Change 20, 121–133 (2015).

[b50] Naro-Maciel E., Mrosovsky N. & Marcovaldi M. A. Thermal profiles of sea turtle hatcheries and nesting areas at Praia do Forte, Brazil. Chelonian. Conserv. Biol. 3, 407–413 (1999).

[b51] Houghton J. D. R. . Protracted rainfall decreases temperature within leatherback turtle (*Dermochelys coriacea*) clutches in Grenada, West Indies: ecological implications for a species displaying temperature dependent sex determination. J. Exp. Mar. Biol. Ecol. 345, 71–77 (2007).

[b52] Kamel S. J. & Mrosovsky N. Nest site selection in leatherbacks, *Dermochelys coriacea*: Individual patterns and their consequences. Anim. Behav. 68, 357–366 (2004).

[b53] Topp J. M. W. & Sheppard C. R. C. Higher plants of the Chagos Archipelago in Ecology of the Chagos Archipelago (eds Sheppard C. R. C. & Seaward M. R. D.) 225–240 (Linnean Society, 1999).

[b54] NCAR. *International Comprehensive Ocean-Atmosphere Data Set (ICOADS) Release 2.5*. (2015) Available at: rda.ucar.edu/datasets/ds540.1. (Accessed: 8th July 2015).

[b55] Wunderground. *Weather History for FJDG*. (2015) Available at: www.wunderground.com/history/airport/FJDG. (Accessed: 19th July 2015).

[b56] Howard R., Bell I. & Pike D. A. Thermal tolerances of sea turtle embryos: current understanding and future directions. Endang. Species Res. 26, 75–86 (2014).

[b57] Ackerman R. A. The nest environment and the embryonic development of sea turtles in Biology of Sea Turtles (eds Lutz P. L. & Musick J. A.) 83–106 (CRC Press, 1997).

[b58] R Core Team. *R: a language and environment for statistical computing*. (2015) Available at: www.R-project.org. (Accessed: 5th August 2015).

[b59] South A. Rworldmap: a new R package for mapping global data. R J. 3, 35–43 (2011).

