# Supplementary material for: Male hatchling production in sea turtles from one of the world’s largest marine protected areas, the Chagos Archipelago
Source: Sci Rep. 2016 Feb 2;6:20339. doi: 10.1038/srep20339 (PMC4735811; doi:10.1038/srep20339)
Supplement: Supplementary Information [file srep20339-s1.pdf]

Supplementary Information

Male hatchling production in sea turtles from one of the world's largest marine protected areas, the Chagos Archipelago

Nicole Esteban[1,+,*], Jacques-Olivier Laloë[1,+], Jeanne A. Mortimer[2], Antenor N. Guzman[3], Graeme C. Hays[4]

[1] Swansea University, Department of Biosciences, Swansea, SA2 8PP, United Kingdom

[2] University of Florida, Department of Biology, Gainesville, FL 32611, United States of America

[3] US Naval Facilities Engineering Command Far East, Public Works Department, Diego Garcia, FPO AP 96595, British Indian Ocean Territory

[4] Deakin University, Geelong, Centre for Integrative Ecology (Warrnambool campus), Victoria, Australia

*n.esteban@swansea.ac.uk

+these authors contributed equally to this work

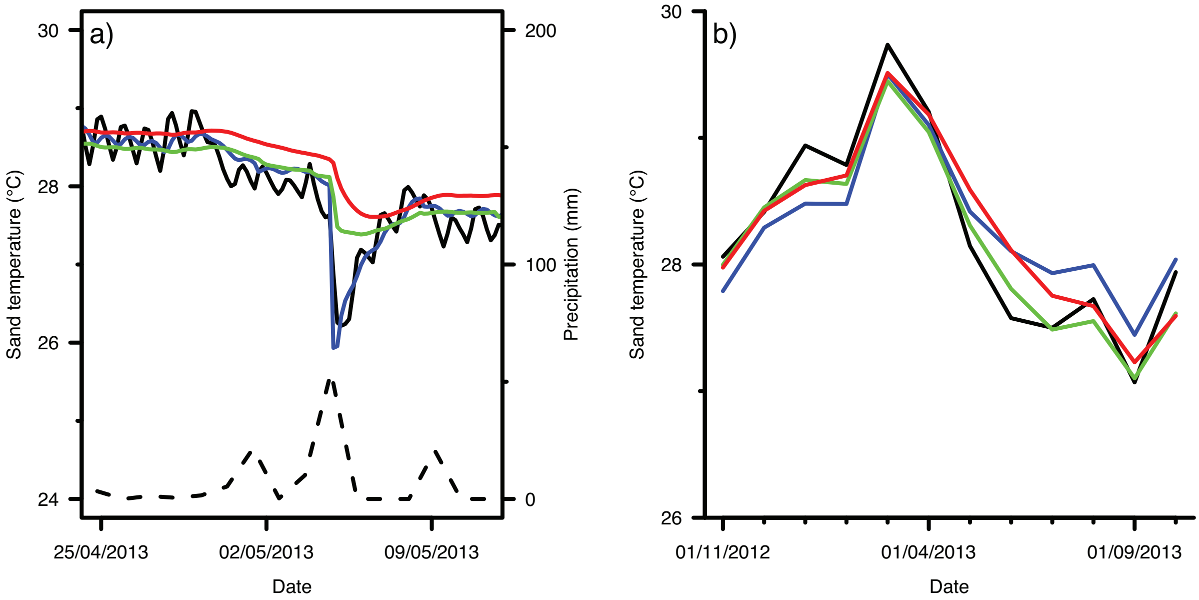

Figure S1. The effect of precipitation and depth on sand temperature. Sand temperatures were recorded at different depths: 30 cm (black line), 50 cm (blue line), 70 cm (green line), and 80 cm (red line). **(a)** Rainfall events coincide with sudden drops in sand temperature. The dashed line represents daily precipitation (secondary y-axis). Data shown are from site 1 for loggers placed in the area partially shaded in vegetation above the Spring HWL. **(b)** The effect of depth on sand temperature is not constant through the seasons and temperatures recorded at shallow depths (in black) are cooler relative to the temperatures recorded at deeper depths (in red) only during cooling periods. This relationship is inversed during warming periods. Data shown are mean monthly sand temperatures for loggers placed in the area partially shaded in vegetation above the Spring HWL.
